# Stronger tuberculosis laboratory networks and services in Africa essential to ending tuberculosis

**DOI:** 10.4102/ajlm.v6i2.519

**Published:** 2017-03-31

**Authors:** Philip C. Onyebujoh, Ajay K. Thirumala, Amy Piatek

**Affiliations:** 1 World Health Organization, Regional Office for Africa, Harare, Zimbabwe; 2 TB Laboratories, Bangalore, India; 3 Global Health Bureau, USAID, Washington, DC, United States

## Introduction

The recent transition from the Millennium Development Goals to the Sustainable Development Goals highlights a major paradigm shift in the global fight against tuberculosis.^1^ The World Health Organization (WHO) End TB Strategy, approved by the World Health Assembly in 2014, calls for a 90% reduction in tuberculosis deaths and an 80% reduction in tuberculosis incidence rates by 2030, compared with 2015.^2^ Globally, tuberculosis death rates have dropped by 22%, and an estimated 49 million lives were saved between 2000 and 2015.^3^ In Africa, since 2000, the downward trends in tuberculosis prevalence, incidence and death rates are notable. Between 1990 and 2012, the Central African Republic, Egypt, Eritrea, Ghana, Malawi, Niger, Rwanda and Uganda were some of the best-performing countries, recording reductions of more than 50% in all three indicators. By contrast, Cameroon, Equatorial Guinea, Lesotho, Liberia, Mauritania, Sierra Leone, South Africa and Swaziland all more than doubled their 1990 rates for at least two of the above tuberculosis indicators.^4^

**FIGURE 1:**
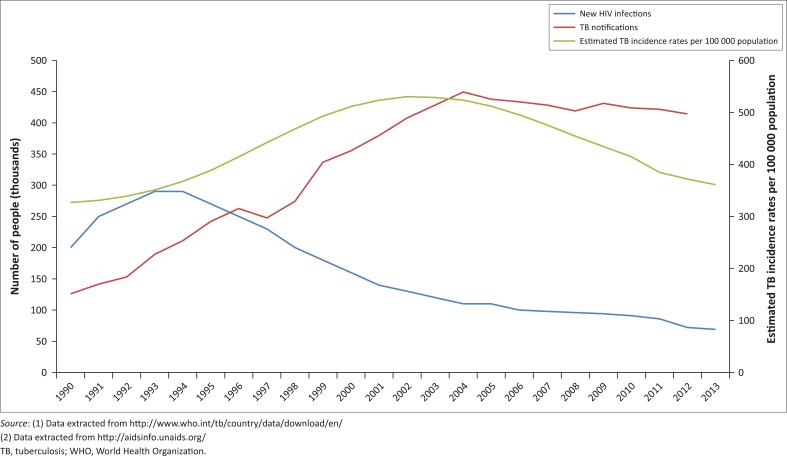
Tuberculosis incidence and notification rates. Estimated tuberculosis incidence rates per 100 000 population and tuberculosis notifications reported to WHO (1) are presented, along with new HIV infections reported to the UNAIDS database (2) between 1990 and 2013 in 18 sub-Saharan African countries (Southern and Eastern Africa, excluding South Africa).

Africa is home to more than a quarter of the estimated incidence of all tuberculosis cases (2.7 million; 26%) and has twice the global estimated incidence rates (239/100 000 people) of new tuberculosis cases.^3^ Tuberculosis death rates in Africa are three times greater than the global average.^3^ Although estimated tuberculosis incidence rates are coming down from incidence rates in 2000 in sub-Saharan Africa, the large number of people living with HIV and the dual epidemic of tuberculosis/HIV challenge efforts to accurately diagnose tuberculosis and treat people suffering from both diseases, reflecting the relatively stable numbers of new tuberculosis case notifications since 2010 (Figure 1). Improving equitable access to tuberculosis diagnosis and treatment remains the most pressing need for Africa where laboratory services play a pivotal role.

## Tuberculosis laboratory services in Africa

Public health laboratory organisational structures and services for the diagnosis of infectious diseases, including tuberculosis, are heterogeneous and suffer from several challenges in Africa.^5,6^ Assessment of the strengths, weaknesses, opportunities and threats regarding tuberculosis laboratory services provide possibilities for objective interventions at both the country and the regional level in Africa (Table 1).

Efforts to diagnose tuberculosis in Africa are lagging behind estimated incidence of the disease, as reflected in the stable case detection rates for the last five years (Table 2). In 2015, 48% of estimated incident tuberculosis cases (all forms) were detected and national tuberculosis control programmes were notified, which is significantly lower than the 61% global average.^3^ New tuberculosis case notifications have decreased since 2009 in line with decreases in estimated tuberculosis incidence rate and tuberculosis/HIV incidence rates. Of the cases notified, 64% were bacteriologically confirmed, reflecting the challenge in the capacities of national tuberculosis control programmes to enable universal access to accurate molecular tuberculosis diagnostics, ensuring notification and prompt treatment to all those people who are diagnosed with tuberculosis.

New strategies and policies to guide diagnostic interventions have been introduced in Africa, in line with the global tuberculosis control policies of the WHO. Increasing investments are being made by national governments and donor agencies to accelerate efforts for preventing, diagnosing, and treating tuberculosis, although large gaps remain in meeting need.^3^ Since 2011, investments in molecular tuberculosis diagnostics have multiplied many fold, as reflected by deployment of an increasing number of Cepheid GeneXpert^®^ systems and modules across Africa.^7^ However, these efforts were not being translated into increased diagnosis of tuberculosis cases or increased laboratory confirmation of cases that were notified. Data reported to the WHO by the Ministries of Health in the African Region, indicate that the numbers of laboratories providing tuberculosis diagnostic services using smear microscopy and GeneXpert have gradually increased from 2009. Between 2009 and 2014, the number of microscopy laboratories increased from 10 501 to 15 233 (45%). Furthermore, the number of laboratories able to diagnose tuberculosis with GeneXpert increased from zero to 817 GeneXpert systems (18 735 GX modules) since it was introduced for the first time in 2011 (Table 3). However, the overall number of new laboratory-confirmed tuberculosis cases has not increased. As a more sensitive and accurate molecular diagnostic tool, the potential of the GeneXpert technology to improve diagnosis of tuberculosis/HIV cases has yet to be fully realised with regard to the overall increase in the number of HIV-positive tuberculosis cases detected.

**TABLE 1:** Key strengths, weaknesses, opportunities and threats for tuberculosis laboratory services in Africa.†

Strengths	Weaknesses
Over 15 000 tuberculosis microscopy laboratories in public settings; 78 tuberculosis culture and DST laboratories; 61 LPA laboratories (at the end of 2015)	High tuberculosis / HIV rates and mortality; Increasing MDR tuberculosis rates and mortality
Rapid expansion of molecular technologies; GeneXpert: 871 GX systems; 18 735 GX modules	Weak laboratory organizational structures; Quality laboratory systems; Networks and accreditations
Three SRLs (South Africa, Uganda, Benin); Plans for expansion	‘Missed-to-diagnose’ cases for tuberculosis, MDR tuberculosis, and tuberculosis/HIV
ASLM strategies and vision driven by strong partnership with USG	Inequitable ‘reach’ of laboratory services / systems
GLI Africa-driven laboratory agenda in the past three year	Sub-optimal up-take of rapid diagnostics; Inadequate funding; Infrastructure
Global Fund-supported regional laboratory networking initiatives	Inadequate diagnosis strategies for the key and vulnerable populations
Young technical personnel; Availability and low human resources hiring costs	Inadequate data on population dynamics of some key and vulnerable groups
Strong and well supported NTPs for tuberculosis care (Southern Africa)	Low funding and poor ownership of laboratories by MoH and changing partners
Emerging local and regional leadership for tuberculosis/HIV, MDR-tuberculosis	Low commitment from health personnel; Inadequate career path and retention plans
High partner support (technically strong); Long standing relationships	Short-term strategy / plans of partner funds (typically 2 years or less)
Regional strategies; Country NSPs; WHO-AFRO missions for NTPs	Unclear service pathway for tuberculosis laboratories in MoH organogram
Existing community systems with NGOs / CSOs	No clear plans / funding for maintenance of laboratory equipment on purchase
Strong scientific evidence for programmatic tuberculosis interventions	Absence of integrated laboratory-specific strategic plans; No adequate capacity for universal tuberculosis DST provision
Increasing focus on tuberculosis surveillance / reporting systems	Lack of standardised reagents and equipment sources available for laboratories to utilise
Increased accreditation and training support through ASLM	Lack of comprehensive electronic surveillance systems for laboratories
Increased awareness of gaps in tuberculosis laboratory services in NTP	

**Opportunities**	**Threats**

Streamlining laboratory organisation structures	Lack of adequate financial resources in lower income countries
Networking laboratories at regional levels	Socioeconomic determinants as risk factors for tuberculosis (poverty, stigma, etc.)
Networking laboratory professional bodies across the region	Overdependence on development partners / donor support
Trainings for developing a highly skilled, motivated laboratory workforce	Poorly coordinated partner technical support, sometimes overlapping purposes
Integrating parallel systems; Avoiding wastage of resources	Inadequate strategic planning for utilisation of existing resources
High impact strategies for tuberculosis (miners, transient-urban and cross-border migrants, KAP)	Donor exhaustion and quick changes in partner priorities
Innovative strategies for community role to enhance equitable access	Occasional political and social weaknesses and disturbances
Integrated systems with focus on point-of-care diagnostics / rapid diagnostics	
Enhanced disease monitoring surveys / surveillances in general	
Collaboration between donors and technical / implementation partners	

ASLM, African Society for Laboratory Medicine; CSO, civil society organisations; DST, drug susceptibility testing; GLI, Global Laboratory Initiative; GX, GeneXpert; KAP, knowledge, attitudes and practises; LPA, line probe assay; MDR, multi-drug resistant; MoH, Ministries of Heath; NGO, non-governmental organization; NTP, National Tuberculosis Program; NSP, National Strategic Plan; SRL, Supranational Reference Laboratory; USG, United States Government; WHO-AFRO, World Health Organization, Regional Office for Africa.

† , Compilations are based on the WHO-AFRO-IST (Inter-country Support Team for East/Southern Africa) Tuberculosis programme review mission reports, country-laboratory assessments reports, and periodic meeting reports of GLI- Africa (2013–2016). Authors acted as external reviewers in several such missions.

**TABLE 2:** New tuberculosis and relapse cases notified in Africa, 2010–2015.†

Year	Total new and relapse tuberculosis case notifications	Case detection rate (all forms)	Proportion of new and relapse pulmonary tuberculosis cases bacteriologically confirmed
2010	1 380 530	50%	64%
2011	1 393 544	50%	57%
2012	1 353 513	49%	59%
2013	1 337 693	49%	57%
2014	1 303 327	48%	62%
2015	1 296 122	48%	64%

*Source*: Data extracted from World Health Organization tuberculosis database: https://extranet.who.int/tme/generateCSV.asp?ds=notifications

† , Data from 47 WHO African Region countries. Data from Equatorial Guinea for 2013, 2012 and Comoros for 2015 and 2010 are not included.

**TABLE 3:** Tuberculosis laboratory services in Africa, 2009–2014.

Year	Total no. of laboratories		No. of new, laboratory-confirmed tuberculosis cases	No. of HIV-positive tuberculosis cases	Total no. of laboratories		No. of laboratory-confirmed cases of RR- or MDR-TB
	Smear microscopy	GeneXpert systems			Drug susceptibility testing (phenotypic)	Line probe assay	
2009	10 501	-	639 238	370 245	65	19	11 239
2010	11 855	-	750 221	394 332	68	34	18 826
2011	12 800	121	683 104	466 075	67	36	14 786
2012	13 612	318	670 390	456 187	64	55	29 553
2013	13 861	618	591 882	444 385	76	56	31 387
2014	15 233	871†	635 356	415 657	78	61	25 653

*Source*: Data extracted from World Health Organization tuberculosis database. Available from: https://extranet.who.int/tme/generateCSV.asp?ds=labs

MDR, multi-drug resistant; RR, rifampicin resistant.

† , Includes 18 735 GX modules in total for 871 GX systems; MDR, multi-drug resistant; RR, rifampicin resistant.

**TABLE 4:** Regional distribution of first- and second-line tuberculosis drug susceptibility tests performed for notified cases reported in 2015 in Africa.

WHO AFRO sub-region	No. of countries	No. of new laboratory-confirmed TB cases	Tested for DST/GX No. (%)	RR/MDR TB detected No. (%)	No. of MDR-TB cases detected	No. of RR/MDR-TB cases on treatment No. (%)	No. of MDR-TB patients on treatment who received SL-DST No. (%)	XDR-TB detected
Central	7	33 070	1979 (5.98%)	280 (14.15%)	227	128 (45.71%)	81 (63.28%)	0
North	2	10 771	749 (6.95%)	71 (9.48%)	53	50 (70.42%)	NA	NA
East	7	124 845	26 323 (21.08%)	1533 (5.82%)	628	1404 (91.59%)	151 (10.75%)	0
West	17	125 020	33 203 (26.56%)	1811 (5.45%)	771	1118 (61.73%)	103 (9.21%)	2 (1.9%)
SADC	14	341 939	287 181 (83.99%)	21 959 (7.65%)	9580	13 734 (62.54%)	3563 (25.94%)	551 (15.4%)

**Total**	**47**	**635 645**	**349 435 (54.97%)**	**25 654 (7.34%)**	**11 259**	**16 434 (64.06%)**	**3898 (23.72)**	**553 (14.1%)**

AFRO, Regional Office for Africa; DST, Drug susceptibility testing; GX, GeneXpert; MDR, multi-drug resistant; RR, rifampicin resistant; SADC, Southern African Development Community; SL, second-line; TB, tuberculosis; WHO, World Health Organization; XDR, extensively drug-resistant.

Eight African countries, including Angola, the Democratic Republic of Congo, Ethiopia, Kenya, Mozambique, Nigeria, South Africa and Zimbabwe, are included on the WHO’s list of 30 multi-drug resistant (MDR), high tuberculosis-burden countries.^3^ It is necessary that Africa develop tuberculosis culture and drug susceptibility testing (DST) laboratories (for first- and second-line anti-tuberculosis medicines) capable of treatment monitoring and diagnosis. The rapid implementation of GeneXpert technology in Africa has resulted in an increase of laboratory-confirmed cases of rifampicin-resistant tuberculosis, a surrogate for MDR tuberculosis, of 128% between 2009 and 2014 (Table 4). During the same period, the total number of laboratories providing DST also increased by 20%, and the number of laboratories offering line-probe assays increased by 221%. Providing universal access to tuberculosis DST remains a newly-defined target of the End TB Strategy. Universal access to DST is currently defined as DST for at least rifampicin among all patients with bacteriologically-confirmed tuberculosis, and further DST for at least fluoroquinolones and second-line injectable agents among all tuberculosis patients with rifampicin resistance.

**FIGURE 2:**
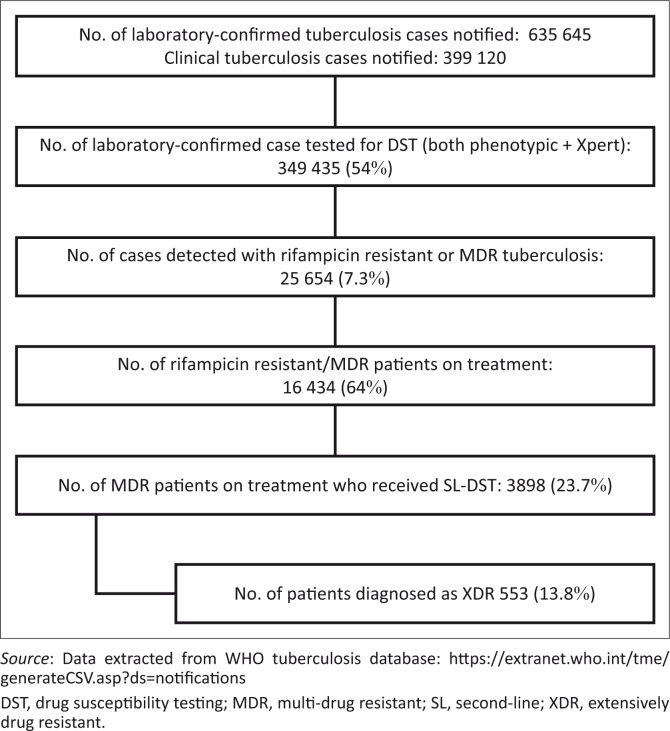
Tuberculosis drug susceptibility testing. First- and second-line tuberculosis drug susceptibility tests performed for notified cases reported in 2015 in Africa.

Preliminary assessment of existing gaps for achieving universal access to DST (Figure 2), based on 2015 case notifications in Africa, indicated that only 349 435 (54%) laboratory-confirmed tuberculosis patients had access to a first-line DST. In 2015, of the 16 434 patients who were diagnosed and treated for MDR tuberculosis, only 3898 (23.7%) could get a second-line DST test. Thus, the challenge that lies ahead for achieving universal access to first- and second-line DST suggests a need to close the 46% gap in access to first-line DST, and the 76.3% gap for second-line DST among tuberculosis and MDR-tuberculosis patients on treatment.

Regional disparities exist in the gaps in access to universal DST, with the southern Africa development community sub-region having better access than the rest of Africa, mainly because of progress made in South Africa proportionate to the number of tuberculosis patients (Table 4). However, these gaps are expected to be bridged through the recent endorsement of rapid molecular line probe assay for second-line drugs and the introduction of shorter-term regimens for programmatic management of drug-resistant tuberculosis.^8^

The pace of establishing tuberculosis diagnostic services needs to be viewed in the context of quality assessment measures and proficiencies for testing. Quality management systems were introduced in laboratory networks in Africa as an integrated effort starting in 2008, and commendable efforts were made through support from the African Society for Laboratory Medicine for Strengthening Laboratory Management Towards Accreditation to the ISO 15189 standard. Tuberculosis-specific quality management roadmaps for accreditation of quality standards were advocated by the Stop TB Partnership’s Global Laboratory Initiative. Most peripheral laboratories in Africa have at least one component of an external quality assessment programme for smear microscopy (usually panel testing). WHO data indicate that less than half of the smear microscopy laboratories performed adequately on external quality assessments (Table 5). Nearly two-thirds of DST laboratories reported meeting WHO-specified quality standards and about half of the laboratories providing Xpert^®^ MTB/RIF test or line probe assay were routinely monitored for quality under programmatic conditions.

**TABLE 5:** Quality of tuberculosis laboratory services in Africa, 2009–2014.

Year	Microscopy laboratories		DST laboratories		GeneXpert laboratories with EQA	LPA laboratories with EQA
	with EQA	with acceptable performance	with EQA	with acceptable performance
2009	50%	40%	71%	63%	**-**	32%
2010	60%	47%	53%	49%	**-**	47%
2011	56%	49%	73%	70%	**-**	28%
2012	65%	44%	81%	80%	24%	73%
2013	70%	49%	68%	67%	53%	63%
2014	69%	44%	64%	63%	55%	46%

*Source*: Data extracted from World Health Organization tuberculosis database. Available from: https://extranet.who.int/tme/generateCSV.asp?ds=labs

DST, drug susceptibility testing; EQA, external quality assurance; LPA, line probe assay.

All of this taken together strongly suggests that available tuberculosis laboratory services need to be optimally utilised in Africa as new technologies are introduced during the next few years. There is an urgent need to redefine the purpose, structure and use of diagnostic networks to diagnose tuberculosis and drug-resistant tuberculosis in an epidemiological context and within the operational capacities of the existing, resource-limited settings of Africa without compromising quality standards.

## Priorities for strengthening tuberculosis services in Africa

### Make use of new ambitious global strategies and goals, and diagnostic testing advances

To accelerate global tuberculosis efforts, the World Health Assembly approved the End TB Strategy^9^ in 2014 as a 20-year strategy with the vision of a ‘world free of TB’ and the goals of ‘zero deaths, disease and suffering due to TB’.^2^ The strategy’s milestones and targets are ambitious: by 2025, there should be a 75% reduction in tuberculosis deaths and 50% reduction in new tuberculosis cases, and by 2035, there should be nearly no deaths from tuberculosis and a 90% reduction in new tuberculosis cases. The Global Plan to End TB 2016–2020 was developed concurrently by the Stop TB Partnership as a costed plan to implement the End TB Strategy and provide a path for policy makers to achieve the Strategy’s milestones, including a roadmap for new diagnostics.^10^ Both the End TB Strategy and Global Plan clearly articulate that countries must design and implement diagnostic services that promote early and rapid diagnosis of tuberculosis and MDR tuberculosis by using new technologies and expanding the pool of people to be tested. It is fully expected that countries will provide universal DST to all people with tuberculosis. Africa must use the opportunities within both the Strategy and Global Plan and translate them into interventions to improve diagnostic services.

Compared to a few years ago, laboratory services in Africa now have better access to advanced tuberculosis diagnostics, including the rapid molecular assays that can accurately diagnose tuberculosis and drug-resistant tuberculosis in less than a day, improved sensitivity for microscopy through fluorescent light-emitting diode technology and faster automated liquid tuberculosis culture systems. Recent advancements in diagnostic tests to detect HIV, including advanced point-of-care rapid diagnostic tests, early infant diagnostic tests and viral load tests, are all critically important in Africa as part of the continuum of care for people co-infected with tuberculosis and HIV.

Laboratory quality management and accreditation systems are now available, including the Strengthening Laboratory Management Towards Accreditation and Stepwise Laboratory Improvement Process Towards Accreditation tools developed especially for Africa.^11^ Other enabling e-tools for tuberculosis diagnostics are increasingly made available, including: software platforms that transfer diagnostic outcomes and testing information in real-time to tuberculosis programme officers, clinicians and health workers; commercial specimen transport solutions that can reduce the potential for culture contamination; and mapping technology to allow the visualisation and overlay of all diagnostic networks in a country and the ability to optimise integrated networks in response to existing and future diagnostic algorithms.

### Strengthen strategic policy, planning and health system components to improve tuberculosis diagnostic networks

The data above suggest that increasing the number of laboratories may improve access to tuberculosis diagnostic services to an extent. However, it may not necessarily increase overall tuberculosis case notifications under programmatic conditions. Enabling interventions such as appropriate changes in national diagnostic policies and algorithms, adequately resourced ambitious strategic plans, increased training for human resources, efficient sputum transport systems to avoid unnecessary diagnostic- and patient-delayed testing, and increased participation of community level organisations are all needed. The need for early access to point-of-care diagnostic services and treatment monitoring is greatest at the primary healthcare level and at the community level, whereas more sophisticated extensive diagnostic capacity is needed at regional- or central-level facilities.^12^ Suboptimal linkages with clinical services in many countries often result in either no diagnosis or diagnoses without treatment initiation or delayed treatment, which in turn lead to poor treatment outcomes, including death. It is expected that with increased electronic connectivity between new and emerging molecular diagnostic tools and treatment centres, the diagnostic-treatment gap will diminish significantly and clinical outcomes will improve.

### Integrate public health laboratory and diagnostic networks

To address the deficiencies in laboratories’ diagnostic capacity, the integration of public health laboratory networks and surveillance systems^13^ for infectious diseases remains an urgent requirement. This need became clear during the recent Ebola outbreak in West Africa, which had a devastating socioeconomic impact on the countries affected,^14^ as well as on their public health systems.^15^ Well-designed, integrated systems would also meet the need for the implementation of the International Health Regulations^16^ and be in line with the Global Health Security Agenda with its current efforts to promote global health security as an international priority.^17^ Integrating laboratory services under one roof would be enhanced by networking with adjunct public health institutions, disease-specific referral laboratories and centres of excellence, facilitating systematic engagement of all governmental and non-governmental health structures. It is envisaged that linking the capacity to detect and manage MDR and extensively-drug-resistant tuberculosis to both the Global Health Security Agenda and the recently forged Declaration on Combating Antimicrobial Resistance will strengthen control of MDR and extensively-drug-resistant tuberculosis globally.

Strengthening collaborative tuberculosis/HIV activities (i.e., an integrated approach for prevention, diagnosis, and treatment among co-infected patients) will remain critical in addressing the tuberculosis/HIV epidemic in sub-Saharan Africa. Integrated laboratory services under one roof have the potential to reduce repeated visits of patients to health facilities by comprehensively screening for multiple infectious diseases, thus improving early case detection and management, in addition to optimising the use of laboratory resources. The fast-growing transition toward more non-communicable conditions in most settings can be tackled through integrated service provision. By default, several laboratory facilities at the district level in Africa provide ‘integrated services’, in one form or other, due to resource constraints and sharing of facilities and personnel. The challenges are mainly related to inadequate infrastructure, equipment, skilled laboratory personnel and their mentoring and supervision, resulting in inadequate integration. The integrated approach demands systematic training and recruitment of high-quality and skilled laboratory scientists, technologists and other laboratory personnel. To achieve universal health coverage, as set out in the End TB Strategy, interdependent, fully-integrated laboratory networks at the country and regional levels are needed, with a minimum set of diagnostic technologies for key infectious diseases prevalent at district and subdistrict levels.

### Establish more regional collaboration

A persuasive way to enhance diagnostic capacity is through the establishment of regional laboratory networks. The capacity of national reference laboratories for culture and drug resistance testing in many African countries has been developed through several multi-country collaborative efforts, such as the EXPANDx-TB project and the East African Laboratory Network, established by the World Bank and aimed at setting up cross-cutting laboratory services with a clear surveillance focus. These networks have been playing an increasingly critical supportive role in the diagnosis and management of drug-resistant tuberculosis.

The strengthening of laboratory systems should be further facilitated through new African regional collaborative initiatives across intergovernmental health bodies and communities. Regional health networks have an important role in facilitating adoption and adaptation of global policies and allowing better care and surveillance through increased domestic funding commitments within the region. The East Central Southern African Health Community regional tuberculosis laboratory networking supported by the Global Fund is one example of the right direction in this regard.

### Prioritise research to inform African-specific solutions

Key components of the End TB Strategy are the early and rapid diagnosis of tuberculosis, universal DST, and systematic screening of contacts and people who practise high-risk activities.^16^ Research across the continuum from basic to new tool development and operational research is an essential requirement of the End TB Strategy and should be strengthened, especially in Africa. The existing laboratory and diagnostic systems at various levels in Africa would greatly benefit from increased resource mobilisation for, and participation in, the development of new tuberculosis point-of-care diagnostics, shorter treatment regimens and, ultimately, an effective vaccine by 2025. While new rapid diagnostic technology has become increasingly available, further scale up must be informed through well-designed operational assessments, including the integration of testing platforms across diseases and with diagnostic algorithms employed in other diseases. African-specific operational research is needed to improve many components of the diagnostic network, including linkages from diagnostic to treatment centres, ideal testing algorithms for country-specific settings, expanding skills and capacities of laboratory technologists, operational requirements for technologies at the facility level, such as quality electricity supply, adequate logistics for diagnostic commodity procurement and distribution within the country, effective integrated sample transport networks and timely feedback of results.

### Garner commitment and leadership from within African governments

While concerted action from all stakeholders is needed, national governments must demonstrate stronger political will and provide stewardship. Efforts must be enhanced to ensure that diagnosis and treatment systems for tuberculosis and drug-resistant tuberculosis are available and well aligned across the different levels of their public health systems. Lower-level laboratories must be linked to higher-level laboratories for access to follow-up testing for efficient patient management, ensure optimal use of different technologies at different levels of the tiered network, and maintain staff competence in these techniques.^12^ Ministries of Health should ensure that national medical laboratory strategic plans – either as stand-alone documents or as part of national tuberculosis strategic plans – are put in place and implemented. Strategic oversight at the country level should continuously ensure the effective management of laboratories, the quality of testing, and the efficient use of the network’s tuberculosis diagnostic services.

### Conclusions

Tuberculosis laboratory services and networks suffer multiple deficiencies in Africa. Implementation of rapid molecular diagnostic laboratory tools for tuberculosis in the past five years, has resulted in an increased number of people diagnosed with and treated for drug-resistant tuberculosis in Africa, although drug-sensitive tuberculosis cases and tuberculosis/HIV cases have not seen increased case notifications, primarily due to restricted diagnostic algorithms, and capacity and access limitations. There is an urgent need to redefine the purpose, structure and optimal use of diagnostic networks to diagnose tuberculosis within the resource-constrained setting of Africa and tuberculosis epidemiology. Well-planned and -resourced national strategies are needed to achieve the laboratory targets set forth by the Global Plan to strengthen services toward ending tuberculosis. Appreciable efforts made in the introduction of new laboratory technologies in the last five years in several countries need to be multiplied at all service delivery levels to maximise technology utilisation to close the diagnostic gap in providing universal access to tuberculosis DST by 2020 in Africa. Integration of laboratory services within public health institutions and public–private collaborations would improve tuberculosis case detection. Tuberculosis is preventable, treatable and curable, and governments need to swiftly demonstrate strong leadership in ending tuberculosis as a leading infectious disease in Africa.
